# Stiff limb syndrome with lower limb myoclonus: A case report

**DOI:** 10.1097/MD.0000000000018160

**Published:** 2019-12-10

**Authors:** Chang-Guo Zhang, Lan-Lan Li, Yao-Yao Feng, Jing Chen

**Affiliations:** Department of Neurology, The Third People's Hospital of Huzhou, Zhejiang Province, China.

**Keywords:** electromyogram, gAD-ab, stiff limb syndrome, stiff-man syndrome

## Abstract

**Rationale:**

stiff limb syndrome (SLS) is a variant of stiff-man syndrome, primarily affecting a specific limb. Its diagnosis has always been challenging due to the lack of a specific confirmation test. We present a rare case of a patient with lower limb myoclonus and rigidity.

**Patient concerns:**

A 53-year-old male presented with a sudden onset of progressive left lower extremity myoclonus and muscle rigidity for 3 days. He rapidly showed signs of right lower limb involvement with severe joint stiffness and inability to walk.

**Diagnosis:**

The symptoms nature, physical examination, careful elimination of differential diagnosis suggested a diagnosis of stiff limb syndrome.

**Interventions:**

Intravenous infusion of gamma globulin 0.4 mg/kg coupled with baclofen and clonazepam were given after admission. He also received an injection of botulinum toxin A to relieve his muscle stiffness.

**Outcomes:**

The patients’ condition improved after the initial treatment with complete disappearance of muscle twitching. Further improvements were seen later on after the local administration of botulinum toxin A.

**Lessons:**

Stiff limb syndrome shares the same complex symptoms with many other conditions. Its diagnosis relies heavily on clinical presentations and on ruling out other conditions. However, unusual symptoms such as myoclonus can occur in few cases and together with the rarity of the condition, the prevalence of misdiagnosis is high. Therefore, being aware and recognizing the signs and symptoms is crucial for proper management. Additionally, EMG is a very important test if the present condition is suspected. However, a negative EMG result or a negative anti-glutamic acid decarboxylase antibody test should not exclude SLS diagnosis.

## 1 Introduction

Stiff-man syndrome is a rare neurological immune disease characterized by truncal muscles fluctuation, progressive stiffness, and paroxysmal painful spasms.^[1–3]^ It is currently considered to be a spectrum disorder comprised of the classical stiff-man syndrome and other variants such as stiff limb syndrome, paraneoplastic stiff-man syndrome, and progressive encephalomyelitis with tonic-clonic phases.^[1,4]^ The primary cause is often thought to be associated with high levels of anti-glutamic acid decarboxylase antibody (GAD-Ab), glycine receptor antibodies, and paraneoplastic antibodies.^[5]^ Treatments usually include immunosuppressants (immunoglobulin, plasma exchange) and muscle relaxants such as clonazepam and baclofen.^[6]^ We report a case of rapid onset of Stiff-man syndrome with myoclonus.

## 2 Case report

A 53-year-old male patient presented with symptoms of progressive left lower extremity myoclonus and muscle rigidity that started 3 days ago. The left lower extremity myoclonus and stiffness started suddenly without any apparent cause with a frequency of once every few minutes. They significantly worsened on the second day with an increase in frequency of once every few seconds and persisted even during sleep. After admission and on the fourth day, similar symptoms started to appear on the patient's right lower limb. Those symptoms were accompanied by a severe joint stiffness and the inability to straighten it, to stand or walk. The Patient denied any prior history of chronic conditions such as diabetes mellitus, Grave disease or similar symptoms among his family members. He also denied any prior surgery.

The physical examination indicated a bilateral muscle twitching of lower extremities, an elevated muscle tone, a positive knee reflex (++++) and positive bilateral Babinski sign. The electromyography results displayed signs of motor unit discharge. Meanwhile, the brain, cervical, thoracic, and lumbar spine MRI scans were all negative. Paraneoplastic antibodies, GAD-65, GAD-67 antibody, and glycine receptor antibody were all tested and reported negative. Additionally, the chest CT and PET-CT scans were also negative.

The patient was given an intravenous infusion of gamma globulin 0.4 mg/kg with additional symptomatic treatments such as baclofen and clonazepam on the fourth day after admission. No obvious adverse reaction was observed after the initial treatment. He showed signs of remission after 5 days of treatment with total disappearance of his muscle twitching. However, no significant changes in muscle stiffness were seen after the initial treatment and the patient was still unable to walk or stand.

Two months after the onset of the condition and after a thorough discussion with the patient, he received injections of botulinum toxin A in both lower limbs partially alleviation his muscle stiffness. Additionally, he was able to stand and walk but with a spastic gait.

Informed consent was obtained from the patient for the description, data utilization, and publication of this report.

## 3 Discussion

This patients’ symptoms were of sudden onset with an acute progressive course. The stiffness and painful spasms were localized to the lower extremities and are indicative of pyramidal tract involvement. Several conditions such as classical stiff-man syndrome, stiff limb syndrome (SLS), Neuromyotonia and so on, can present in a similar manner. However, the fact that the symptoms were localized to the lower extremities and the patients’ responsiveness to the initially given therapeutic combination are supportive of SLS.^[7]^ Unlike classical stiff-man syndrome, SLS mainly affects distal extremities and often starts from the foot upward.^[7,8]^ It does not affect the paraspinal and abdominal muscles.^[9]^ Interestingly, this patient also had myoclonus-like twitching on his left lower extremity, a presentation that is not usually seen in SLS. This alone makes ruling out progressive encephalomyelitis with rigidity and myoclonus (PERM) paramount. Even so, the patient did not show additional symptoms such as ataxia, eye movement abnormalities, and dizziness indicating brain stem involvement. Additionally, due to the fact that PERM is a serious, life-threatening autoimmune disease characterized by rigidity, muscle pain and spasm, sensory abnormalities, symptoms related to the brainstem and spinal cord, autonomic dysfunctions, dyspnea, and spontaneous stimulation-induced myoclonus, symptoms that are absent in our case, it is safe to say that the likelihood is remote. Another condition that needs to be ruled out is Neuromyotonia or ISSAC’ syndrome.^[10,11]^ Patients with such condition often have symptoms of muscle twitching or fiber twitching. In this condition, a typical electromyography result would be indicative of repeated, spontaneous, and multiform motor unit potentials with myoclonic and myotonic discharges.^[12–15]^ Patients with such condition would have a positive neuron voltage gate antibody test.^[12,13]^ The electromyogram findings of our current patient are not indicative of such changes, and the VCKC antibody test came negative.

Previous studies have indicated that 80% of immune SPS patients are positive for anti-glutamate decarboxylase antibody (GAD-ab), while 11.9% are positive for anti-glycine receptor antibody.^[16–18]^ Another study reported a case of SLS with leukemia and positive anti-glycine receptor antibody.^[19]^ Interestingly, in this case, all tests related to commonly screened antibodies such as serum and cerebrospinal fluid paraneoplastic antibodies, and anti-glycine receptor antibodies were found to be negative.^[19]^ We simultaneously screened for 2 subtypes of GAD antibodies (GAD65 and GAD67). We believe that pathways required for GABA synthesis might have been affected indicating the potential involvement of more antibodies in the pathophysiology of this condition. However, the lack of a positive antibody test further supporting the initial diagnosis made it even more challenging.

Electromyogram (EMG) is crucial for the diagnosis of SLS with positive ratios of 72%, 85.7%, and 87.5% for immunological, paraneoplastic, and cryptogenic SPS, respectively.^[20]^ Therefore, EMG should be routinely performed on all suspected SLS patients. Additionally, negative EMG results or GAD antibodies cannot rule out SLS diagnosis,^[8]^ and long term follow-up and assessment are needed to monitor disease progression.

This patient was finally diagnosed with immune SLS based on an extensive elimination of differential diagnosis and received a treatment comprised of immunoglobulin, baclofen, clonazepam, and others. Although his muscle twitching was resolved, he still exhibited signs of rigidity in the lower extremities. At present, new therapeutic options such as spinal cord stimulation,^[21]^ dantrolene, intrathecal injection of baclofen, cannabis products have been tried for the treatment of SPS.^[20]^ In this case, the local injection of botulinum toxin A has achieved satisfactory results in improving the patient's quality of life. Further follow-up is required.

## Author contributions

**Conceptualization:** ChangGuo Zhang, Yao-Yao Feng.

**Data curation:** Lan-Lan Li, Yao-Yao Feng, Jing Chen.

**Investigation:** Jing Chen.

**Methodology:** ChangGuo Zhang.

**Validation:** Lan-Lan Li.

**Writing – original draft:** ChangGuo Zhang.

**Writing – review & editing:** Lan-Lan Li, Jing Chen.
